# Impact Toughness of Subzones in the Intercritical Heat-Affected Zone of Low-Carbon Bainitic Steel

**DOI:** 10.3390/ma11060959

**Published:** 2018-06-06

**Authors:** Zhenshun Li, Xuemin Zhao, Dongri Shan

**Affiliations:** 1 Engineering Training Center, Qilu University of Technology (Shandong Academy of Sciences), Jinan 250353, China; zxm@qlu.edu.cn; 2 School of Mechanical and Automotive Engineering, Qilu University of Technology (Shandong Academy of Sciences), Jinan 250353, China; shandongri@126.com

**Keywords:** intercritical heat-affected zone, impact toughness, martensite-austenite constituent, critical size, low-carbon bainitic steel

## Abstract

The subzones of the intercritical heat-affected zone (IC HAZ) of low-carbon bainitic steel were simulated by using a Gleeble-3500 simulator to study the impact toughness. The results showed that the IC HAZ is not entirely brittle and can be further divided into three subzones according to the impact toughness or peak welding temperature; the invariant subzone heated between the critical transformation start temperature (*A*_c1_) and 770 °C exhibited unchanged high impact toughness. Furthermore, an extremely low impact toughness was found in the embrittlement subzone, heated between 770 and 830 °C, and the reduction subzone heated between 830 °C and the critical transformation finish temperature (*A*_c3_) exhibited toughness below that of the original metal. The size of the blocky martensite-austenite (M-A) constituents was found to have a remarkable level of influence on the impact toughness when heated below 830 °C. Additionally, it was found that, once the constituent size exceeds a critical value of 3.0 µm at a peak temperature of 770 °C, the IC HAZ becomes brittle regardless of lath or twinned martensite constitution in the M-A constituent. Essentially, embrittlement was observed to occur when the resolved length of initial cracks (in the direction of the overall fracture) formed as a result of the debonding of M-A constituents exceeding the critical Griffith size. Furthermore, when the heating temperature exceeded 830 °C, the M-A constituents formed a slender shape, and the impact toughness increased as the area fraction of the slender M-A constituents decreased.

## 1. Introduction

Low-carbon bainitic steels with relatively high strength and toughness have been developed to improve weldability, weight reduction, and energy saving capability. They are widely used in the construction of important structures and facilities such as offshore drilling platforms, naval vessels, and mining hydraulic support [1,2]. In addition to having the sufficient strength to withstand external pressure, these steels must possess high impact toughness to assure safety in the event of unexpected accidents, as they are often implemented in complex service environments. However, the microstructures and mechanical properties of the steels can be significantly altered by the thermal cycles of welding; this consequently results in poor toughness in the heat-affected zone (HAZ). The intercritically reheated coarse-grained heat-affected zone (IC CG HAZ), or intercritical heat-affected zone (IC HAZ), has the least toughness because of the presence of martensite-austenite (M-A) constituents prior to the austenite grain boundaries. Such regions are usually considered as local brittle zones [3,4,5]. In previous studies, the IC CG HAZ, or IC HAZ, was determined via thermal simulation because of its small size. Specimens were usually heated to a predetermined peak temperature between the critical transformation start and finish temperatures or *A*_c1_ and *A*_c3_, respectively. As an example, a simulated IC HAZ would correspond to one small point in the actual welded IC HAZ, as its microstructure varied throughout the zone due to experiencing different heating temperatures ranging from *A*_c1_ to *A*_c3_. More specifically, considering the entire IC HAZ as a brittle zone based on the result of one point is not accurate. Research on the subzones remains insufficient, particularly research on the ends of the IC HAZ.

The mechanism by which M-A constituents affect the impact toughness is still unclear. Several studies have reported that the amount of M-A constituents is the primary metallurgical factor affecting the impact properties [6,7,8]. Li and Baker identified correlations between the toughness and fraction of M-A constituents [9]. However, Kaplan reported that the correlation between the toughness and total fraction of M-A constituents can be somewhat weak; it was found that this correlation can be improved by focusing on the fraction of M-A constituents that are massive and large in size [10]. Davis reported that the embrittlement of the HAZ is not only related to the amount of M-A constituents but also to the particular microstructure, i.e., a near-connected grain boundary network of blocky M-A particles [11]. Luo stated that slender M-A constituents are more detrimental to toughness than massive ones [12]. Conversely, Li concluded that both massive and slender M-A constituents lead to the formation of cracks and decrease the impact toughness [13]. The microstructure of M-A constituents can be rather complicated. Hrivnak found that M-A constituents consist of lath martensite, plate martensite, cementite, and a small portion of retained austenite [14]. Mohseni reported that the embrittlement of the IC HAZ is closely related to the presence of twinned martensite in the M-A constituents [15]. In this study, IC HAZs were simulated with different peak temperatures by using a Gleeble-3500 simulator to comprehensively study the impact toughness of different subzones and better understand the properties of the IC HAZ. The results can be used to evaluate the impact toughness of the HAZ in actual welding joints.

## 2. Materials and Methods

An 800 MPa grade low-carbon bainitic steel plate with a thickness of 25 mm was used in this study. The plate was produced by implementing a thermomechanical control process and high-temperature tempering. The chemical composition was 0.07 wt % C, 0.34 wt % Si, 1.65 wt % Mn, 0.04 wt % Nb, 0.06 wt % V, 0.25 wt % Cr, 0.14 wt % Ni, and 0.13 wt % Mo, with the remaining content being Fe. Specimens with dimensions of 11 × 11 × 55 mm^3^ were cut from the middle of the plate along the rolling direction. The welding thermal cycle simulation was conducted in a Gleeble 3500 thermomechanical simulator (DSI, Austin, TX, USA). A Pt-10% Rh thermocouple was welded to the middle of the specimens to record the temperature, and a quartz dilatometer was fixed onto the same section to record the dilatation. The critical transformation start and finish temperatures of the experimental steel were first measured at a heating rate of 150 °C·s^−1^, with the results identifying *A*_c1_ and *A*_c3_ as 758 and 913 °C, respectively. The specimens were heated to different peak temperatures (760, 765, 770, 800, 830, 860, 890, or 910 °C) for a duration of 1 s before being cooled to 100 °C. The heating rate for the thermal cycles was 150 °C·s^−1^, and the cooling time between 800 and 500 °C (*t*_8/5_) was 11 s. When the peak temperature was less than 800 °C, the cooling time was adjusted to generate the same portion of the cooling curve that would have been produced under the condition that the pre-cooling temperature was above 800 °C. The specimens were machined into standard Charpy V-notch specimens. They were cooled to −20 °C in a low-temperature thermostat (CDW-60, SUNS, Shenzhen, China) then subjected to impact loading on an impact tester (JB30B, TIMES, Beijing, China) within 2 s. Five specimens were tested for each heating and cooling condition. Samples etched into the LePera etchant proposed by Ale [16] were examined by using optical microscopy. Image-Pro PLUS analysis software (6.0, Media Cybernetics, Rockville, MD, USA) was used to quantitatively examine the metallography of the specimens. The fracture surfaces of Charpy specimens were observed by using scanning electron microscopy (SEM) (QUANTA200, FEI, Hillsboro, OR, USA). Transmission electron microscopy samples were prepared to obtain a detailed analysis of the microstructure. The specimens were thinned and perforated in a twinjet electro polisher (TenuPol-5, Struers, Ballerup, Denmark) under the condition of immersion in an ethanol-based solution with 8.3% perchloric acid. Thin foil samples were observed with TEM (TECNAI G2F30S-TWIN, FEI, Hillsboro, OR, USA) operating at 200 kV.

## 3. Results

**Figure 1:**
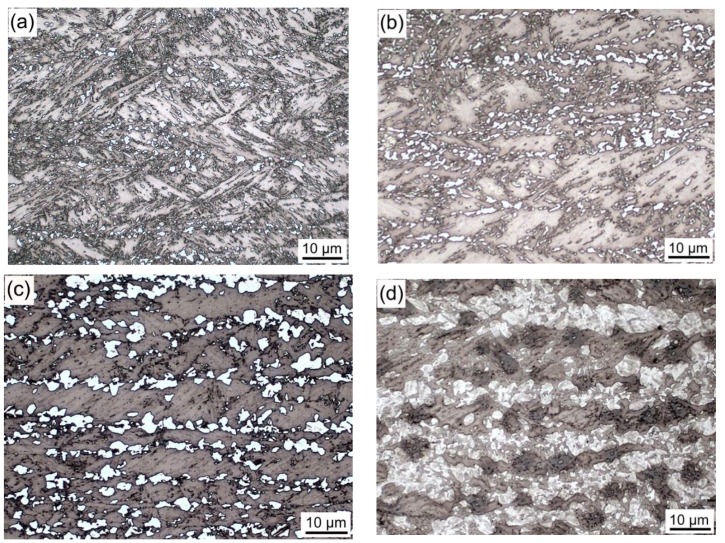

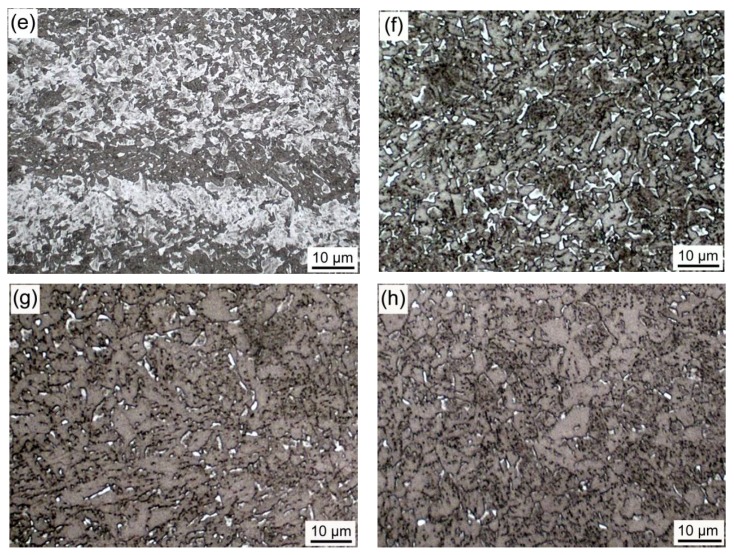
Effect of peak temperature on the microstructures of the IC HAZ: (**a**) 760, (**b**) 765, (**c**) 770, (**d**) 800, (**e**) 830, (**f**) 860, (**g**) 890, and (**h**) 910 °C.

Optical micrographs of simulated IC HAZs heated at various peak temperatures are presented in Figure 1. The original microstructure of the experimental steel was lath bainite. The prior austenite grain boundaries ran nearly parallel to the rolling direction. Furthermore, the M-A constituent could be distinguished from the matrix because of its white color. Small blocky M-A constituents started to emerge at the grain boundaries when the specimen was heated to 760 °C. The size and fraction of these constituents were observed to increase with increased peak temperatures. At 830 °C, single M-A constituents could not be distinguished, and a semi-continuous layer of M-A constituents covered the grain boundaries. When the peak temperature exceeded 830 °C, the microstructures of the IC HAZ were primarily granular bainite, and the M-A constituents became slender in shape and uniformly distributed in the matrix. The fraction was observed to decrease with increased peak temperatures.

**Figure 2:**
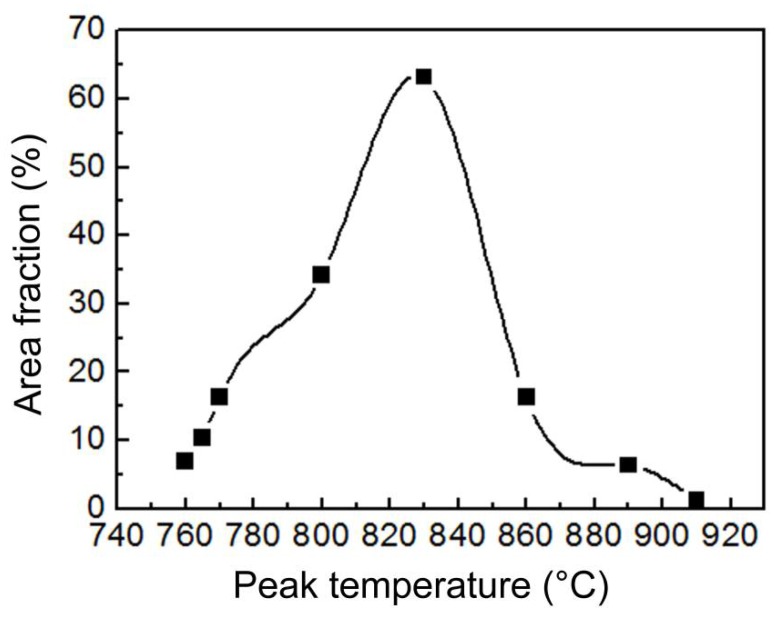
Area fraction of the M-A constituents in the IC HAZs as a function of peak temperature.

**Figure 3:**
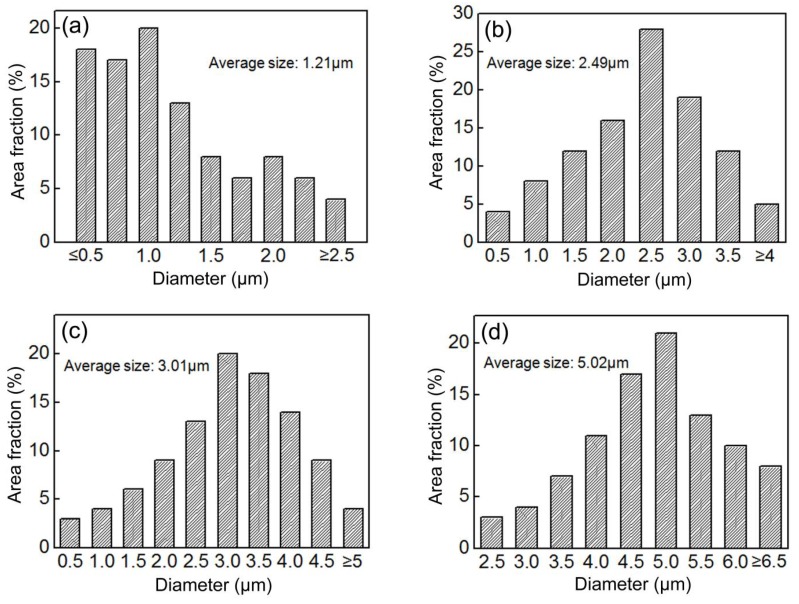
Size distribution of the blocky M-A constituents in the IC HAZ for different peak temperatures: (**a**) 760, (**b**) 765, (**c**) 770, and (**d**) 800 °C.

As is shown in Figure 2, the area fraction of the M-A constituents increased with increasing peak temperatures up to 830 °C. Beyond this temperature, the area fraction decreased with further increases in temperature. Figure 3 shows the size distributions of the blocky M-A constituents in the IC HAZ heated to various peak temperatures below 830 °C. The blocky M-A constituents were approximated to be globes, and their diameter was measured. Note that the difference between the most probable size (corresponding to the maximum area fraction) and average size was quite small for all heating temperatures. The size of the blocky M-A constituents was found to increase with increasing peak temperatures, following a trend similar to that of the area fraction.

**Figure 4:**
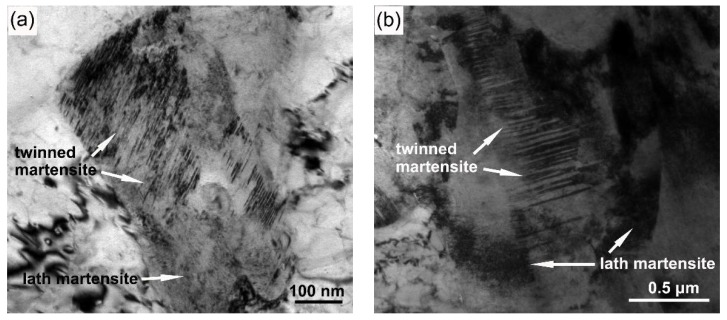

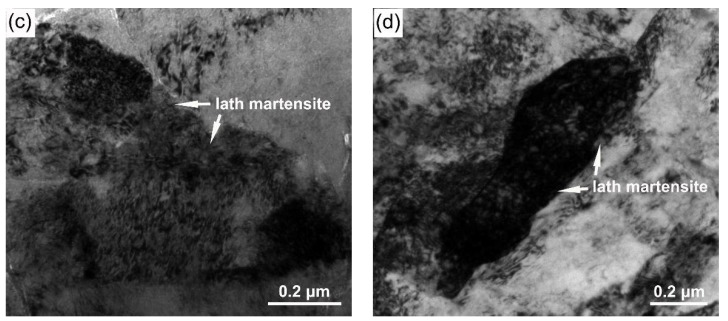
TEM images of M-A constituents in the IC HAZ with varying peak temperatures: (**a**) 760, (**b**) 770, (**c**) 800, and (**d**) 860 °C.

Figure 4 shows typical TEM images of the M-A constituents in the IC HAZ. For peak temperatures of 760 and 770 °C, the M-A constituents comprised twinned and lath martensite. Because twinned martensite has higher carbon content than lath martensite, it can be assumed that the rapid heating and cooling conditions caused the carbon in the transformed austenite to be uneven. When the peak temperature reached 800 °C, the twinned martensite was no longer present. Alternatively, lath martensite was found in the M-A constituents when the specimens were heated to 800 °C and above.

**Figure 5:**
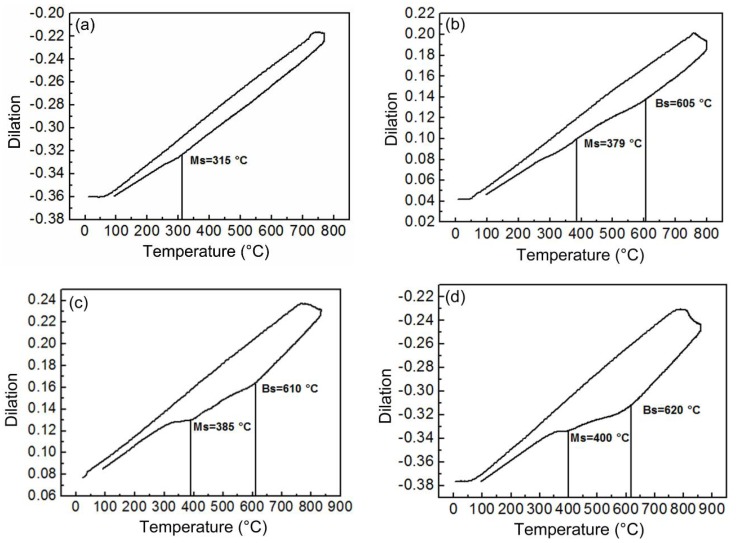
Dilatometric curves for the simulated IC HAZ specimens with varying peak temperatures: (**a**) 770, (**b**) 800, (**c**) 830, and (**d**) 860 °C.

The phase transition processes of the IC HAZ were recorded in the form of dilatation curves, as is illustrated in Figure 5. This figure clearly showed that the M-A constituents comprising twinned and lath martensite were the products of martensite transformation. When the peak temperature was below 800 °C, only martensite transformation could be detected during cooling. This means that the austenite formed during heating was entirely transformed into M-A constituents. For peak temperatures of at least 800 °C, the bainite transformation could be detected before martensite. Additionally, the measured *M*_s values_ of the martensite transformation were 315, 379, 385, and 400 °C, corresponding to peak temperatures of 770, 800, 830, and 860 °C, respectively. Because the value of *M*_s_ is inversely proportional to the carbon content [17], the M-A constituents exhibited decreasing carbon content as the peak temperature increased. The constitution changed from a combination of twinned and lath martensite to solely lath martensite when the peak temperature was 800 °C (Figure 4).

**Figure 6:**
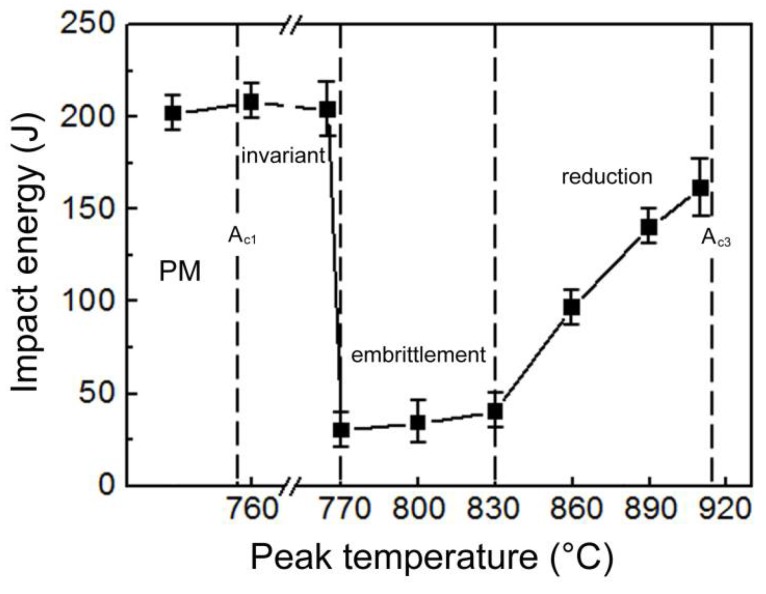
Effect of the peak temperature on the impact toughness of the IC HAZ.

Figure 6 shows the Charpy impact energies of IC HAZs with varying peak temperatures. Minimal deviation from the impact toughness of the parent metal (PM) was observed when the specimens were heated to 760 and 765 °C. However, increasing the peak temperature to 770 °C resulted in an abrupt and sharp decrease in the impact toughness of the specimen, suggesting the onset of IC HAZ embrittlement, which was sustained up to a temperature of 830 °C. A further increase of the peak temperature to above 830 °C corresponded to considerable increase in the impact toughness. As shown in Figure 2 and Figure 6, the relationship between the impact toughness and area fraction of the M-A constituents was not clear when the M-A constituents were blocky and heated below 830 °C. Conversely, the following relationship was quite apparent when the M-A constituents were slender and heated above 830 °C: the impact toughness increased as the area fraction of the M-A constituents decreased.

**Figure 7:**
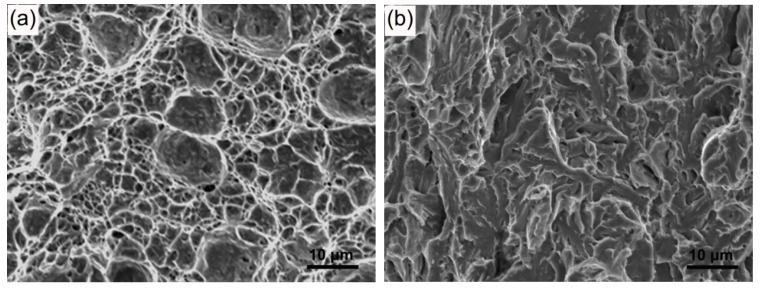

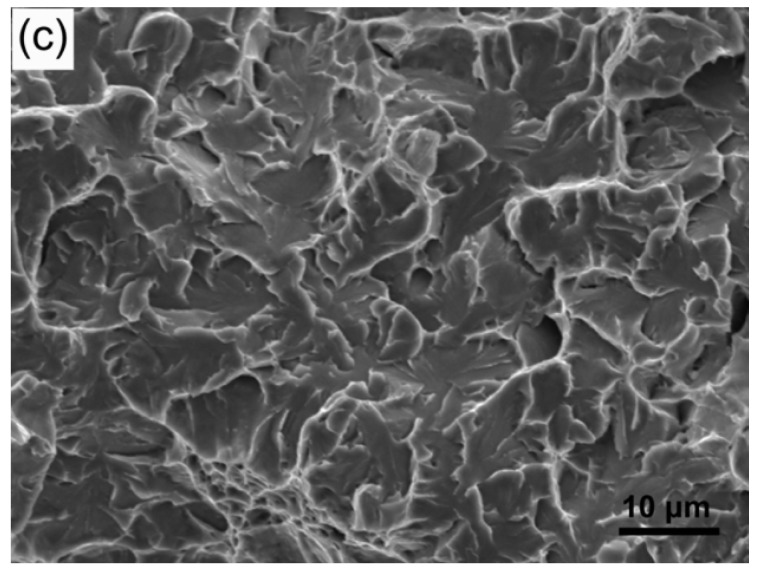
SEM fractographs of the simulated IC HAZ heated to different peak temperatures: (**a**) 765, (**b**) 770, and (**c**) 860 °C.

The appearance of fractures in the IC HAZ validated the results of the impact test. Figure 7a shows that a dimpled fracture occurred at a peak temperature of 765 °C; this observation suggests high impact toughness of the IC HAZ. Brittle cleavage fracture morphology was observed when the peak temperature was 770 °C because of IC HAZ embrittlement. Numerous cracks or voids extending in various directions were also observed for this temperature, as is shown in Figure 7b. Under the condition of a peak temperature of 860 °C, cleavage was present; however, the fracture surface was relatively smooth and fewer cracks and voids were observed, as seen in Figure 7c.

## 4. Discussion

The rapid heating and cooling processes in welding typically result in higher carbon contents in austenite than those resulting from an equilibrium transformation [18,19]. Austenite formed at peak temperatures below 800 °C completely transform to martensite (i.e., M-A constituents) during cooling, as is illustrated in Figure 5a. The high carbon content can induce the production of twinned martensite. Additionally, the additional presence of lath martensite is related to the uneven distribution of carbon in austenite for a limited diffusing time (Figure 4a,b). Furthermore, as the peak temperature is increased, the size and fraction of the M-A constituents increased with the increasing amounts of transformed austenite. However, the decreasing carbon content of austenite causes a portion of the bainite to form prior to the martensite transformation, as is demonstrated by the dilatometric curves (Figure 5b–d). During bainite transformation extra carbon will be injected into the residual austenite, which can be modified to form M-A constituents in response to carbon enrichment [20]. For this reason, the size and fraction of M-A constituents are observed to not only increase. For example, when the temperature exceeds 830 °C, most of the transformed austenite becomes granular bainite because of the low carbon content; this reduces the size and fraction of the M-A constituents transformed from residual austenite. The M-A constituents are distributed at the edges of the granular bainite, forming a slender shape. In spite of the carbon enrichment, the M-A constituents have a significantly higher *M*_s_ at peak temperatures of at least 800 °C, as is shown in Figure 5. Consequently, only lath martensite is produced.

**Figure 8:**
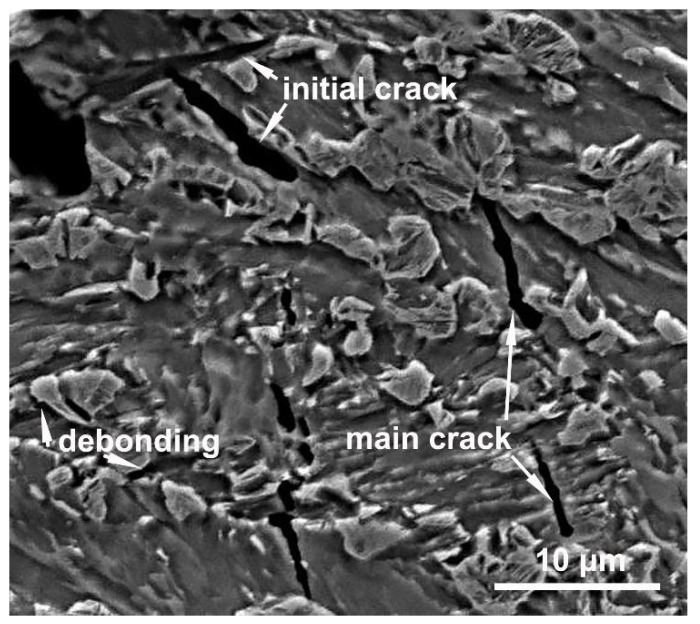
Secondary cracks near the fracture surface; peak temperature = 770 °C.

Because the martensite structure of M-A constituents cause them to have a greater hardness than the matrix, stress concentration will be induced at the interface produced under loading [21]. Consequently, the M-A constituents are debonded from the matrix, as is shown in Figure 8; this finding is consistent with the reports of many previous studies [14,22]. The debonding creates microvoids or cracks with dimensions similar to those of the M-A constituents because the interfaces have been weakened by carbon segregation [23]. Previous studies have proposed that microvoids continuously elongate to become a critical Griffith flaw and induce brittle fractures [24,25]. However, in this study, the continuous elongation of microvoids was retarded in the toughened matrix, forming a blunt tip, as shown in Figure 8. Thus, microvoids can be gradually spheroidized during growth. The subsequent overall growth and coalescence of voids induce dimple fracturing, as is shown in Figure 7a. This implies that small microvoids cannot grow to induce a cleavage fracture. Furthermore, the impact test results indicate that the detrimental effects of M-A constituents with a size of less than 3 µm on the impact toughness of IC HAZ can be ignored. Although small M-A constituents cause stress concentrations and act as potential sites for cracks, the effects of small M-A constituents on impact toughness may have been overstated.

For larger blocky M-A constituents, Davis proposed a model in which the debonding initiates cracks with sizes similar to that of the M-A constituents, which run parallel to the main cracks at the interface [11]. Thus, there is a critical size for the M-A constituents such that a controlled cleavage fracture is produced. Kaplan suggested that this critical size is approximately 2 µm [10]. In this study, however, the actual debonding position at the interface could not be determined, and the directions of initial crack propagation were considered to be random (Figure 8). This randomness may be attributed to the multi-directional stress applied to the M-A constituents, the irregular shapes of the M-A constituents, and the varying extents of interface weakness. The results demonstrated that brittle cleavage fracture is only initiated immediately after the resolved length of the initial cracks (in the direction of the overall fracture) reaches a critical Griffith size. Since embrittlement of the IC HAZ began occurring when the peak temperature reached 770 °C, using Figure 3c as a reference, the critical size of the corresponding M-A constituent has been suggested to be the most probable size (3.0 µm), which is larger than Kaplan’s suggestion. The intersection of initial cracks produces deep steps on the surface of a fracture because of their angled directions relative to the main cracks, as is shown in Figure 7b. Although the detrimental effects of M-A constituents had previously been ascribed to the twinned martensite structure [15,26], it was found that the effect of the martensite structure was negligible for an expanded heating temperature range. Lath martensite was also observed to induce embrittlement of the IC HAZ, even under the condition of reduced carbon content at high peak temperatures. This phenomenon may be due to the low deformability of martensite, including lath martensite, in M-A constituents. Additionally, the size of the blocky M-A constituents is conclusively a significant factor which influences impact toughness, regardless of the martensite structure, because once the size exceeds the critical value of 3.0 µm at a peak temperature of 770 °C, the IC HAZ becomes brittle. However, note that the area fraction of M–A constituents is also presumed to play an important role in the impact toughness because the fractures were observed to have multiple sources (Figure 7b).

In comparison, slender M-A constituents have a higher deformability because of the decreased carbon content, as indicated by the high *M*_s_. This has been reported to result in a higher toughness of the HAZ [27]. Thus, stress concentrations at the interface can be relieved somewhat at the initial stage of loading. At this stage, the impact toughness of the IC HAZ heated to 860 °C was relatively high. The M-A constituents will crack with further loading because of the low hardness and thickness, which can be inferred from the relative smooth fracture surface shown in Figure 7c. As was similarly reported in many previous studies [9,12], the impact toughness was found to increase as the area fraction of the M-A constituents decreased when specimens were heated to a temperature above 830 °C, thereby confirming the increased impact toughness of the IC HAZ.

## 5. Conclusions

(1) The IC HAZ can be further divided into three subzones according to its non-uniform impact toughness. The invariant subzone, heated between *A*_c1_ and 770 °C, has an unchanged high impact toughness. The embrittlement subzone, heated between 770 and 830 °C, exhibits an extremely low impact toughness. The reduction subzone heated between 830 °C and *A*_c3_ has a toughness that is less than that of the original metal.

(2) When the peak temperature is below 830 °C, the size of the blocky M-A constituents is an important factor that affects the embrittlement of the IC HAZ regardless of the martensite structure. Blocky M-A constituents with a size of less than 3.0 µm have no detrimental effect on the impact toughness; however, once the size exceeds the critical value of 3.0 µm, embrittlement of the IC HAZ occurs. Conversely, when the peak temperature exceeds 830 °C, the impact toughness increases as the area fraction of slender M-A constituents decreases.

(3) The debonding of blocky M-A constituents initiates cracks which are angled to the main cracks. Embrittlement of the IC HAZ occurs when the resolved length of the initial cracks exceeds the critical Griffith value.
